# Prognostic role of cyclin B1 in solid tumors: a meta-analysis

**DOI:** 10.18632/oncotarget.13653

**Published:** 2016-11-26

**Authors:** Chenyang Ye, Ji Wang, Pin Wu, Xiaofen Li, Ying Chai

**Affiliations:** ^1^ Cancer Institute (Key Laboratory of Cancer Prevention and Intervention, National Ministry of Education, Provincial Key Laboratory of Molecular Biology in Medical Sciences), Second Affiliated Hospital, College of Medicine, Zhejiang University, Hangzhou 310009, China; ^2^ Department of Thoracic Surgery, Second Affiliated Hospital, College of Medicine, Zhejiang University, Hangzhou 310009, China; ^3^ Department of Surgical Oncology, Sir Run Run Shaw Hospital, College of Medicine, Zhejiang University, Hangzhou 310016, China

**Keywords:** cyclin B1, solid tumors, prognosis, overall survival, meta-analysis

## Abstract

Cyclin B1 is a key mitotic cyclin in the G2-M phase transition of the cell cycle and is overexpressed in various malignant tumors. Numerous studies have reported contradictory evidences of the correlation between cyclin B1 expression and prognosis in human solid tumors. To address this discrepancy, we conducted a meta-analysis with 17 published studies searched from PubMed and Medline. Cyclin B1 overexpression was significantly associated with poor 3-year overall survival (OS) (OR = 2.05, 95% CI = 1.20 to 3.50, *P* = 0.009) and 5-year OS (OR = 2.11, 95% CI = 1.33 to 3.36, *P* = 0.002) of solid tumors. Subgroup analysis revealed that elevated cyclin B1 expression was associated with worse prognosis of lung cancer and esophageal cancer but better prognosis of colorectal cancer. In summary, overexpression of cyclin B1 is correlated with poor survival in most solid tumors, which suggests that the expression status of cyclin B1 is a significant prognostic parameter in solid tumors.

## INTRODUCTION

It is universally acknowledged that dysregulation of the cell cycle is closely correlated with proliferation of cancer cells, and is a hallmark of human carcinomas [1]. Progression of cell cycle is mediated by a series of cyclin-dependent kinases (cdks) and cyclins. Cyclins play vital roles at various phases of the cell cycle by activating specific cdks. Among various cyclin/cdk complexes regulating the cell cycle, cyclin B1/Cdc2 is a widely studied complex, which controls G_2_-M phase checkpoint surveillance, and is essential for initiation of mitosis [2]. Cyclin B1, encoded by the *CCNB1* gene [3], has been demonstrated to play a pivotal role in tumorigenesis and tumor development. In normal conditions, expression level of cyclin B1 is very low and increases sharply only at the G2-M phase transition. Deregulation of cyclin B1 can result in unrestricted cell-cycle progression and malignant transformation [4–7]. Overexpression of cyclin B1 has been reported in various human cancers, including breast [8], colorectal [9], lung [10], prostate [11], pancreatic [12], laryngeal [13], esophageal [14], gastric [15] and hepatocellular [16] cancers. A plenty of studies revealed that cyclin B1 is implicated in the differentiation, growth, apoptosis, metastasis and chemoresistance of cancer cell [14, 17–20]. Given the promoting role of cyclin B1 in tumor development, cyclin B1-targeted prevention and therapy might be beneficial. However, the prognostic merit of cyclin B1 overexpression in various solid tumors is still disputed.

A large number of studies demonstrated that enhanced expression of cyclin B1 in tumor tissue was associated with poor survival of patients with various types of solid tumors such as breast cancer [21, 22], lung cancer [10, 23–25], esophageal cancer [26–28], gastric cancer [15], hepatocellular carcinoma [16], pancreatic cancer [12, 29], embryonal tumors [30], and laryngeal carcinoma [13]. However, other studies revealed that overexpression of cyclin B1 was correlated with favorable outcome of patients with gastric cancer [15] and colorectal cancer [9, 31, 32].

Therefore, we conducted a meta-analysis to assess the prognostic value of cyclin B1 overexpression in human solid tumors. The purpose of this meta-analysis was to evaluate the relevance of elevated cyclin B1 expression with survival in solid tumors, and shed more light on the clinical merit of cyclin B1 as a therapeutic target and prognostic biomarker for solid tumors.

## RESULTS

### Search results and study characteristics

**Figure 1:**
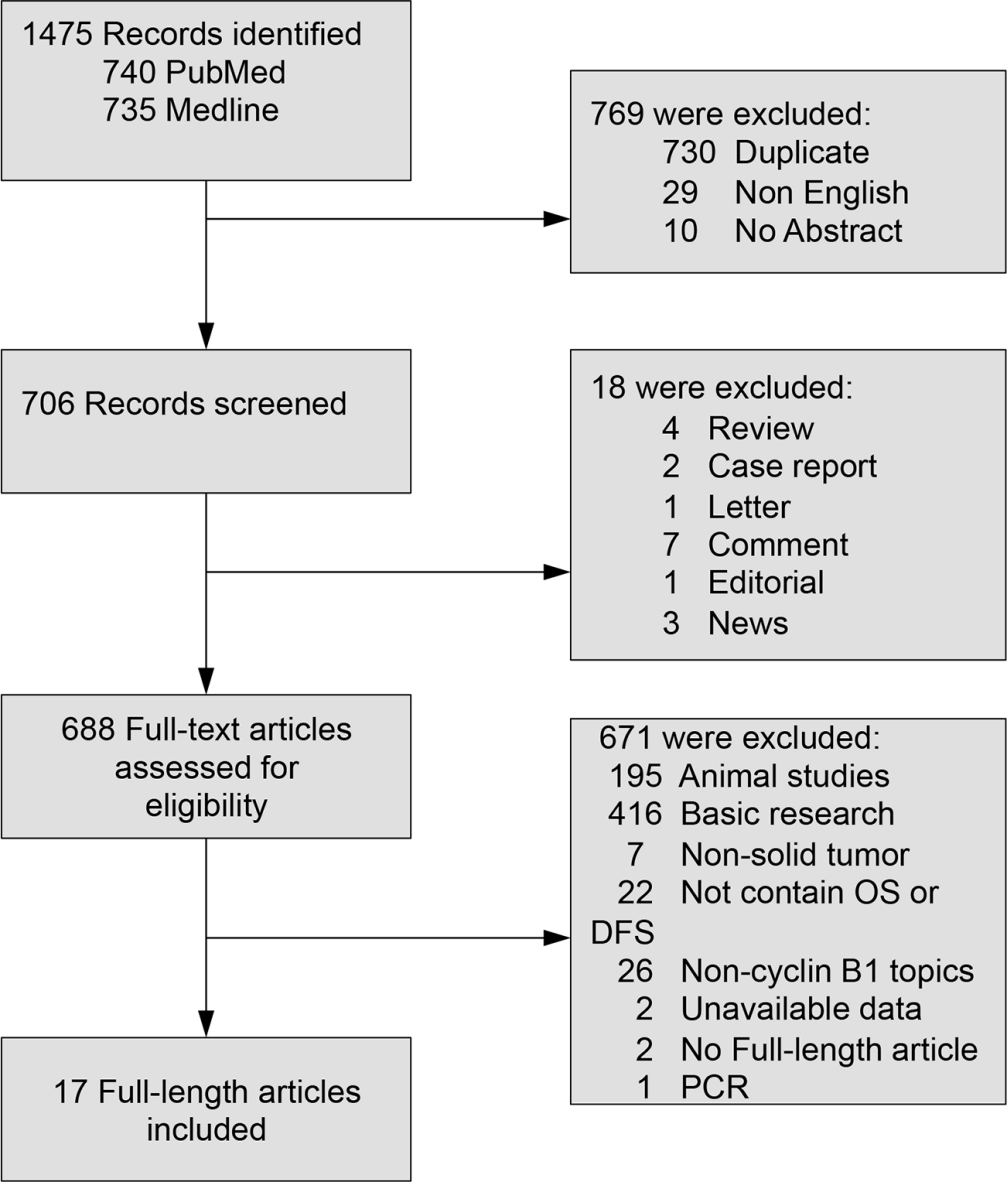
Flow diagram of the meta-analysis process OS: overall survival; DFS: disease-free survival.

17 studies with a total of 2492 patients were included (Figure 1). Characteristics of included studies were shown in Table 1. Four studies evaluated lung cancer [10, 23–25], three evaluated esophageal cancer [26–28], three evaluated colorectal cancer [9, 31, 32], two evaluated gastric cancer [15, 33], and one each evaluated breast cancer [21], hepatocellular carcinoma [16], pancreatic cancer [12], laryngeal squamous cell carcinoma [13] and pediatric embryonal tumors [30]. All these 17 studies evaluated cyclin B1. As for the region, 12 studies were conducted in Asia, 2 studies in America, 2 studies in Europe, and 1 study in Austria.

**Table 1:** Characteristics of studies included in the meta-analysis

References	Country	Type of cancer	Patient No.	Age,median (range)	Male/ Female	Stage	Follow up, months (Range)	Cyclin B1 (+/−) NO.	3–year OS (+/−)%	5–year OS (+/−)%	NOS Score
Arinaga, M., et al. (2003)	Japan	NSCLC	174	65 (35–84)	127/47	I–III	54.6 (1.4–134.6)	74/100	57.44/70.91	48.48/59.05	7
Begnami, M. D., et al. (2010)	Brazil	GC	482	64 (26–84)	308/174	NR	28.3 (0.6–108.6)	229/231	NR	22.6/40	6
Cooper, W. A., et al. (2009)	Australia	NSCLC	90	67 (41–78)	58/32	I–II	68.9 (1–86.7)	52/36	55.89/81.82	44.37/75.02	7
Dong, Y., et al. (2002)	Japan	LC	102	63.49 (38–89)	86/16	I–IV	44.85 (3–60)	40/62	54.75/73.55	54.72/72.13	8
Fang, Y., et al. (2015)	China	CRC	150	NR	88/62	I–IV	NR	88/62	77.31/64.51	65.27/39.83	7
Grabsch, H., et al. (2004)	Germany	CRC	342	68.8 (28–88)	149/181	I–IV	50.4 (5–136.8)	261/69	79.71/75.23	72.67/61.65	7
Huang, T., et al. (2014)	China	EC	105	58.3 (31–83)	65/40	I–III	NR	28/17	67.79/93.68	50.87/87.48	6
Kim, D.-H. (2007)	Korea	GC	23	57.1 (29–77)	15/8	I–IV	68 (3–108)	20/3	88.31/40.11	82.25/40.11	7
Li, J.-Q., et al. (2003)	Japan	CRC	194	NR	104/90	I–IV	NR	68/75	99.02/82.94	NR	7
Moschovi, M., et al. (2011)	Greece	PET	53	NR	28/25	NR	NR	26/16	55.73/84.34	55.74/84.34	7
Nozoe, T., et al. (2002)	Japan	EC	120	65 (36–89)	101/19	I–III	NR	68/52	61.6/85.51	50.7/78.55	7
Soria, J. C., et al. (2000)	USA	NSCLC	77	65	54/23	I	98.4	17/60	41.03/73.37	29.16/59.79	8
Suzuki, T., et al. (2007)	Japan	BC	109	53.1 (23–82)	0/109	I–III	106 (4–157)	46/63	91.67/100	80.15/90.14	8
Takeno, S., et al. (2002)	Japan	EC	71	63.8 (43–84)	63/8	I–IV	NR	35/36	28.14/74.44	21.57/68.99	7
Weng, L., et al. (2012)	China	HC	80	NR	70/10	BCLC stage A	39.0	31/49	/	/	6
Yoshida, T., et al. (2004)	Japan	NSCLC	79	NR	54/25	I–III	NR	13/66	62.82/91.87	62.82/84.99	7
Zhou, L., et al. (2014)	China	PC	241	60 (34–85)	159/82	NR	13 (2–87)	175/66	22.61/41.21	17.29/33.91	7

NSCLC: Non-Small Cell Lung Cancer; GC: Gastric Cancer; CRC: Colorectal Cancer; BC: Breast Cancer; PC: Pancreatic Cancer; HC: Hepatocellular Carcinoma; EC: Esophageal Cancer; OSCC: LC: Laryngeal Carcinoma; PET: Pediatric Embryonal Tumors; NR: Not Reported; NOS: Newcastle–Ottawa Scale; OS: Overall Survival.

### Evaluation and expression of cyclin B1

A depiction of antibodies, detection and definition method, and cut-off values of cyclin B1 used in the included studies was presented in Table 2. Different antibodies were used for the appraisement of cyclin B1 expression by IHC. For anti-cyclin B1 antibody, four studies used clone H-433, three studies used clone 7A9, two studies used clone V152, and eight studies did not report the antibody clone. Among the groups identified as cyclin B1 positive, the median expression of cyclin B1 in solid tumors was 48.78%, range from 16.46% to 86.96%.

**Table 2:** Evaluation of human cyclin B1 by IHC in the selected studies

References	Type of cancer	Cutoff	Antibody (Clone)
Arinaga, M., et al. (2003)	NSCLC	IHC > 10%	anti-cyclin B1;monoclonal antibody (NR); Novocastra
Begnami, M. D., et al. (2010)	GC	IHC > 10%	anti-cyclin B1(V152); DAKO
Cooper, W. A., et al. (2009)	NSCLC	IHC ≥ 5%	anti-cyclin B1(7A9);monoclonal antibody; Novocastra
Dong, Y., et al. (2002)	LC	IHC > 15%	Anti-cyclin B1 (H-433) polyclonal antibody; Santa Cruz
Fang, Y., et al. (2015)	CRC	expression ratio > 3.33	NR
Grabsch, H., et al. (2004)	CRC	IHC > 10%	anti-cyclin B1(7A9);monoclonal antibody; Novocastra
Huang, T., et al. (2014)	EC	IHC score 5–6	anti-cyclin B1(H433); polyclonal antibody; Santa Cruz
Kim, D.-H. (2007)	GC	IHC > 5%	anti-cyclin B1; (NR);Novocastra
Li, J.-Q., et al. (2003)	CRC	IHC ≥ 4.6%	anti-cyclin B1(H-433); Santa Cruz
Moschovi, M., et al. (2011)	PET	IHC > 15%	anti-cyclin B1(7A9); Novocastra
Nozoe, T., et al. (2002)	EC	IHC > 10%	anti-cyclin B1;monoclonal antibody (NR);Novocastra
Soria, J. C., et al. (2000)	NSCLC	IHC ≥15%	anti-cyclin B1; monoclonal antibody (NR);Novocastra
Suzuki, T., et al. (2007)	BC	IHC > 10%	anti-cyclin B1(H-433); polyclonal antibody;Santa Cruz
Takeno, S., et al. (2002)	EC	IHC > 20%	anti-cyclin B1; monoclonal antibody (NR);Novocastra
Weng, L., et al. (2012)	HC	NR	anti-cyclin B1 (V152); monoclonal antibody ;Cell Signaling Technology
Yoshida, T., et al. (2004)	NSCLC	IHC > 15%	anti-Cyclin B1; monoclonal antibody; (NR);Novocastra
Zhou, L., et al. (2014)	PC	IHC grade 2	anti-cyclin B1; monoclonal; Abcam

NSCLC: Non-Small Cell Lung Cancer; GC: Gastric Cancer; CRC: Colorectal Cancer; BC: Breast Cancer; PC: Pancreatic Cancer; HC: Hepatocellular.

Carcinoma; EC: Esophageal Cancer; OSCC: LC: Laryngeal Carcinoma; PET: Pediatric Embryonal Tumors; NR: Not Reported.

### Association of cyclin B1 with survival

**Figure 2:**
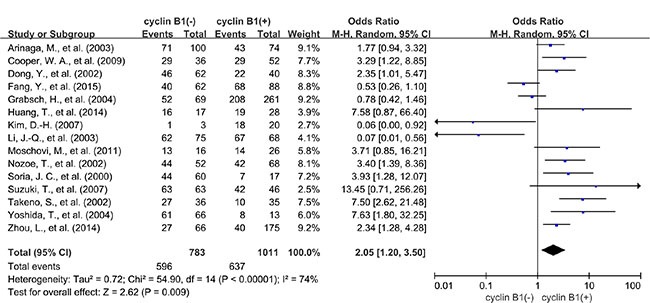
The relationship between expression level of cyclin B1 and 3-year overall survival (OS) of all patients with solid tumors

**Figure 3:**
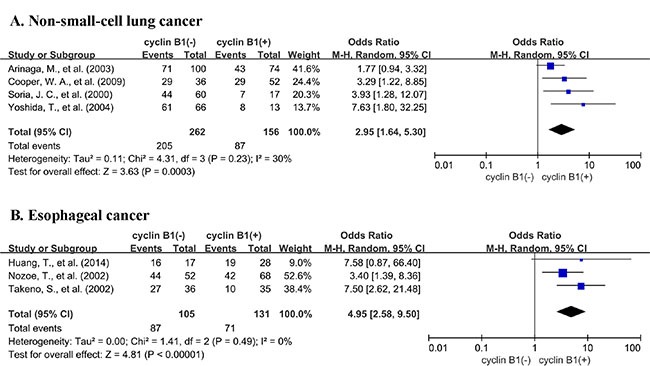
Subgroup analysis of 3-year OS by expression level of cyclin B1 in different cancer types (**A**) non-small-cell lung cancer; (**B**) esophageal cancer.

A total of 15 studies reported data for OS at 3-years. Results showed that cyclin B1 overexpression was associated with worse 3-year OS of solid tumors (OR = 2.05, 95% CI = 1.20 to 3.50, *P* = 0.009) (Figure 2). There was significant heterogeneity among studies (Cochran's Q *P* < 0.00001, *I*^2^ = 74%), so we conducted subgroup meta-analysis to explore whether the heterogeneity was due to different cancer types. 4 studies provided 3-year OS for lung cancer, 3 for esophageal cancer and 3 for colorectal cancer. In the stratified analysis by cancer types, cyclin B1 overexpression was associated with worse 3-year OS of lung cancer (OR = 2.95, 95% CI = 1.64 to 5.30, *P* = 0.0003) (Figure 3A), and esophageal cancer (OR = 4.95, 95% CI = 2.58 to 9.50, *P* < 0.00001) (Figure 3B). However, there was no association between cyclin B1 overexpression and 3-year OS of colorectal cancer (OR = 0.48, 95% CI = 0.21 to 1.14, *P* = 0.10) (Figure S1).

**Figure 4:**
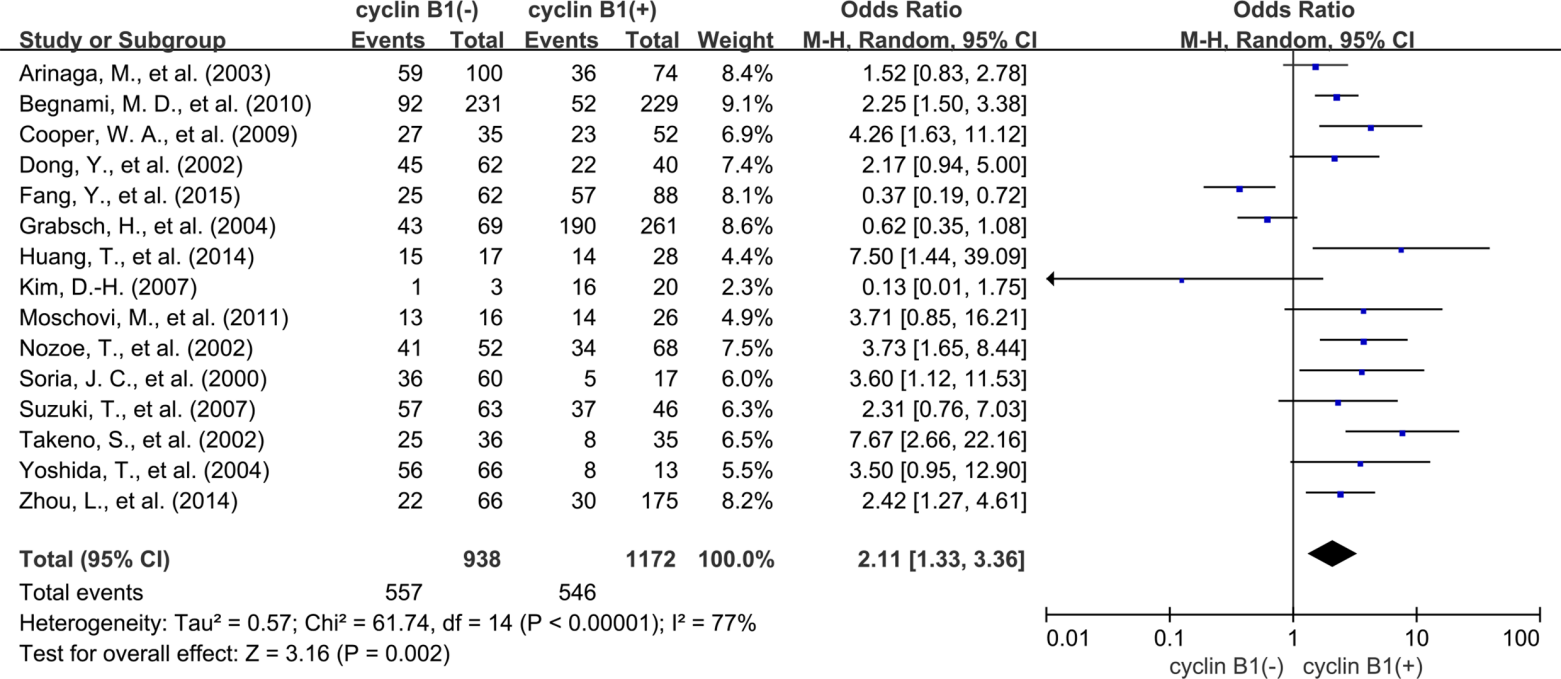
5-year OS by cyclin B1 expression

**Figure 5:**
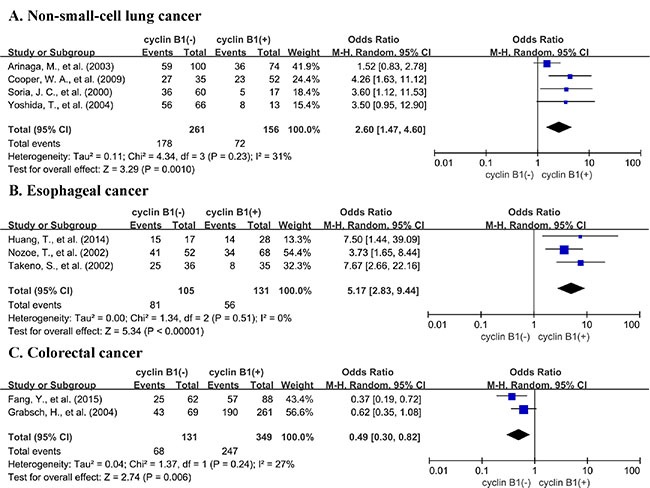
Subgroup analysis of 5-year OS by cyclin B1 expression in various tumor types (**A**) non-small-cell lung cancer; (**B**) esophageal cancer; (**C**) colorectal cancer.

A total of 15 studies reported data for OS at 5-years. Similar to the 3-year OS data, cyclin B1 overexpression was significantly associated with worse 5-year OS of solid tumors (OR = 2.11, 95% CI = 1.33 to 3.36, *P* = 0.002) (Figure 4). There was also high heterogeneity among studies (Cochran's Q *P* < 0.00001, *I*^2^ = 77%), so we performed subgroup meta-analysis according to different cancer types. 4 studies provided 5-year OS for lung cancer, 3 studies for esophageal cancer, 2 for colorectal cancer and 2 for gastric cancer. cyclin B1 overexpression was correlated with worse 5-year OS of lung cancer (OR = 2.60, 95% CI = 1.47 to 4.60, *P* = 0.0010) (Figure 5A) and esophageal cancer (OR = 5.17, 95% CI = 2.83 to 9.44, *P* < 0.00001) (Figure 5B). Interestingly, cyclin B1 overexpression was linked to favorable 5-year OS of colorectal cancer (OR = 0.49, 95% CI = 0.30 to 0.82, *P* = 0.006) (Figure 5C). In addition, there was no association between cyclin B1 overexpression and 5-year OS of gastric cancer (OR = 0.72, 95% CI = 0.05 to 11.51, *P* = 0.82) (Figure S2).

In addition, analysis of 4 studies revealed that there was no association between cyclin B1 overexpression and 10-year OS (OR = 0.97, 95% CI = 0.35 to 2.69, *P* = 0.96) (Figure S3). And meta-analysis of the included studies indicated that expression of cyclin B1 was not significantly associated with 3-year (disease-free survival) DFS (OR = 2.32, 95% CI = 0.90 to 6.00, *P* = 0.08) (Figure S4A) and 5-year DFS (OR = 1.42, 95% CI = 0.69 to 2.90, *P* = 0.34) (Figure S4B). We also assessed the relationship between cyclin B1 overexpression and the TNM stage of solid tumors. The expression level of cyclin B1 was not significantly correlated with TNM stage (OR = 0.51, 95% CI = 0.25 to 1.03, P = 0.06) (Figure S5).

Meta-regression analysis showed that publication year, country, age, gender, and NOS score did not contribute to the heterogeneity (data not shown).

### Sensitivity analyses

Removal of the studies that was an outlier (IRS or IHC < 5%) or no report (NR) with regard to the cutoff of cyclin B1 overexpression by IHC did not affect results for 3- or 5-year OS (OR = 2.24, 95% CI = 1.25 to 4.02, *p* < 0.007; OR = 2.31, 95% CI = 1.47 to 3.64, *P* = 0.0003; respectively). Exclusion of these studies did not substantially reduce heterogeneity for 3- or 5-year OS (Cochran's Q *P* < 0.00001, *I*^2^ = 74%; Cochran's Q *P* < 0.00001, I^2^ = 69%, respectively).

Removal of the studies that patients received adjuvant therapy such as chemotherapy or radiotherapy after curative operation did not influence results for 3- or 5-year OS (OR = 2.11, 95% CI = 1.13 to 3.96, *p* = 0.02; OR = 2.40, 95% CI = 1.42 to 4.04, *p* = 0.001, respectively). Moreover, exclusion of these studies did not reduce heterogeneity for 3- or 5-year OS (Cochran's Q *P* < 0.00001, *I*^2^ = 76%; Cochran's Q *P* < 0.00001, *I*^2^ = 76%, respectively).

Removal of studies with NOS score 6 did not influence results for 3- or 5-year OS (OR =1.94, 95% CI = 1.12 to 3.36, *p* = 0.02; OR = 1.98, 95% CI = 1.15 to 3.40, *p* = 0.01, respectively). Exclusion of these studies did not reduce heterogeneity for 3- or 5-year OS (Cochran's Q *P* < 0.00001, *I*^2^ = 76%; Cochran's Q *P* < 0.00001, *I*^2^ = 79%, respectively).

### Publication bias

Funnel plot analysis indicated that there was no statistical evidence of publication bias in our meta-analysis (data not shown).

## DISCUSSION

In previous cancer studies, overexpression of cyclin B1 was demonstrated to correlate with adverse survival outcome, while some others showed cyclin B1 was a potential biomarker for favorable prognosis. Here we conducted the first systematic evaluation of the literatures with respect to cyclin B1 expression and clinical outcomes in cancer patients to date. We thoroughly assessed survival data of 2492 solid tumor patients in 17 different studies and proved that the expression of cyclin B1 was a prognostic marker of unfavorable clinical outcome, with consistent results of OS at 3- and 5-years. Among the tumor types evaluated, elevated expression of cyclin B1 in tumor tissues was related with worse 3- and 5-year OS of lung cancer and esophageal carcinoma. However, elevated expression of cyclin B1 was associated with better 5-year OS of colorectal cancer. Our study also found there was no significant correlation between cyclin B1 overexpression and OS of gastric cancer. One study reported that cyclin B1 overexpression in gastric cancer tissues was associated with favorable OS, while recent research demonstrated that elevated cyclin B1 expression was correlated with reduced survival. These discrepancies suggest that further studies are warranted to elucidate the underling mechanism and role of cyclin B1 in pathogenesis and prognostic value in different tumor types.

Cyclin B1 is a pivotal mitotic cyclin in the G2 and M phases during the cell cycle [2, 34]. It complexes with the active form of Cdc2 to initiate chromosome condensation, breakdown of the nuclear envelope, and assembly of the mitotic spindle [10]. Recent study reported that cyclin B1/Cdc2 phosphorylates and activates the mRNA cap RNMT (RNA guanine-7 methyltransferase) regulatory domain on T77 in G2/M phase of the cell cycle, promoting the capping activity following mitosis [35]. A growing body of research indicates that dysregulated expression of cyclin B1 is a common event in cancer cells. Binding of Cdc2 can result in phosphorylation of other substrates at inappropriate times and uncontrolled proliferation, due to the inactivation of the tumor suppressor p53 [4, 36]. Downregulation of cyclin B1/Cdc2 reactivates and stabilizes the function of p53 in cancer cells [37, 38]. Accoding to previous data, altered expression of cyclin B1 has been detected in a large variety of solid tumors, and gets involved in cells growth, apoptosis and metastasis. Interestingly, studies also revealed that abnormally expressed cyclin B1 could be discerned by the immune system as tumor antigens in early stages of cancer, which is possible to be used for early cancer detection by monitoring the immune responses. [39–41]. Apart from being a promising biomarker, cyclin B1 may also provide a new target for anticancer therapy. One study showed that the diacerein resulted in the viability reduction of chondrosarcoma cells and G2/M cell cycle arrest by cyclin B1/ Cdc2 downregulation [42]. In RNA level, some research performed gene expression analysis by RT-PCR, and indicated that *CCNB1* was strongly correlated with disease recurrence in solid cancer. However, in the protein level, the association between elevated cyclin B1 and clinical prognosis in solid tumors remains controversial. In light of the important role of cyclin B1 in biology mechanism and clinical application, we performed this meta-analysis to evaluate the prognostic merit of cyclin B1 expression in solid tumors.

This meta-analysis study involves several pivotal implications. First, it reveals that cyclin B1 expression is correlated to unfavorable outcome of most solid tumors, which indicates that cyclin B1 may be a promising therapeutic target. Second, it identifies a subgroup of tumors with adverse outcome in lung cancer and esophageal carcinoma, but with favorable outcome in colorectal cancer. Finally, it highlights the potential clinical application of cyclin B1 as a valuable prognostic biomarker.

Several limitations also exist in this meta-analysis. First, some studies reporting negative results may not be published, which inevitably results in publication bias. Second, subgroup analysis for each type of cancer was not feasible in this meta-analysis because some types of cancer failed to contain enough data. Third, the method and cut-off values for evaluating cyclin B1 expression are nonuniform. Lastly, substantial heterogeneity observed among included studies cannot be completely interpreted in spite of the use of appropriate meta-analytic techniques with random-effects models.

In conclusion, our analysis indicates that cyclin B1 expression is associated with unfavorable outcome in most solid tumors, suggesting that cyclin B1 is a valuable prognostic indicator and a potential therapeutic target for solid tumors.

## MATERIALS AND METHODS

This meta-analysis was carried out based on the Preferred Reporting Items for Systematic Reviews and Meta-Analyses (PRISMA) statement [43]. This study was on the basis of analysis and summary of the results of previous published studies; thus the ethical approval was not necessary.

### Search protocol

We conducted a thorough search of Pubmed and Web of Science for studies evaluating expression of cyclin B1 and survival in cancer patients from 1995 to March 2016. The search terms “cyclin B1” and “neoplasms” were used and the results were restricted to human studies of solid tumors. A total of 740 and 735 entries were identified, respectively. Inclusion criteria were the measurement of cyclin B1 by immunohistochemistry (IHC), availability of survival data for at least 3 years, and publication in English. Studies evaluating gene expression of cyclin B1 measured by polymerase chain reaction were excluded. We checked the citation lists of retrieved articles to ensure sensitivity of the search strategy. Study selection was based on the association of cyclin B1 and survival. Inter-reviewer agreement was assessed using Cohen's kappa coefficient. Any disagreements between authors were resolved by consulting a third author until a final consensus was reached.

### Endpoints of interest

The primary endpoints were overall survival (OS) at 3 and 5 years. Tumors were classified by cyclin B1 expression status using cut-offs as defined by each study.

### Data collection process and quality assessment

The following details were independently extracted by two authors (CYY and JW): name of first author, publication year, country, type of cancer, number of patients, median age, gender, time of follow-up, antibody used for the evaluation, technique used to quantify cyclin B1, and cut-off value to determine cyclin B1 positivity. OS data were extracted from the tables or Kaplan-Meier curves for both cyclin B1 negative and cyclin B1 positive group. The studies included in this meta-analysis were all cohort studies. Two authors independently assessed the quality of each included study by Newcastle-Ottawa Scale (NOS) [44]. The studies with 6 scores or more were denoted as high quality studies. A consensus NOS score for each item was achieved by discussion.

### Data synthesis

The relative frequency of OS at 3- and 5-year between cyclin B1 negative and cyclin B1 positive group was presented as an odds ratio (OR) and its 95% confidence interval (CI). Sensitivity analyses were carried out for different analytical methods and cut-offs for defining cyclin B1 expression and NOS scores for quality assessment of included studies.

### Statistical analysis

Data were extracted from the primary publications and analysed by RevMan 5.3 analysis software (Cochrane Collaboration, Copenhagen, Denmark). Estimates of ORs were weighted and pooled using the Mantel–Haenszel random effect model. Statistical heterogeneity was assessed with the Cochran's Q and *I*^2^ statistics. Differences between subgroups were assessed using methods described in Cochrane Handbook for Systematic Reviews of Interventions [45]. Meta-regression analysis was carried out by Stata 12.0 software (StataCorp LP, College Station, TX). All statistical tests were two-sided, and statistical significance was defined as P less than 0.05.

## SUPPLEMENTARY MATERIALS FIGURES


