# Supplementary material for: Prognostic role of cyclin B1 in solid tumors: a meta-analysis
Source: Oncotarget. 2016 Nov 26;8(2):2224–32. doi: 10.18632/oncotarget.13653 (PMC5356794; doi:10.18632/oncotarget.13653)
Supplement: Supplementary file 1 [file oncotarget-08-2224-s001.pdf]

Prognostic role of cyclin B1 in solid tumors: a meta-analysis

Supplementary Materials

Study or Subgroup	cyclin B1(-) Events	cyclin B1(-) Total	cyclin B1(+) Events	cyclin B1(+) Total	Weight	Odds Ratio M-H, Random, 95% CI
Fang, Y., et al. (2015)	40	62	68	88	41.7%	0.53 [0.26, 1.10]
Grabsch, H., et al. (2004)	52	69	208	261	45.0%	0.78 [0.42, 1.46]
Li, J.-Q., et al. (2003)	62	75	67	68	13.3%	0.07 [0.01, 0.56]
**Total (95% CI)**		**206**		**417**	**100.0%**	**0.48 [0.21, 1.14]**
Total events	154		343			

Heterogeneity: Tau² = 0.32; Chi² = 5.12, df = 2 (P = 0.08); I² = 61%
Test for overall effect: Z = 1.66 (P = 0.10)

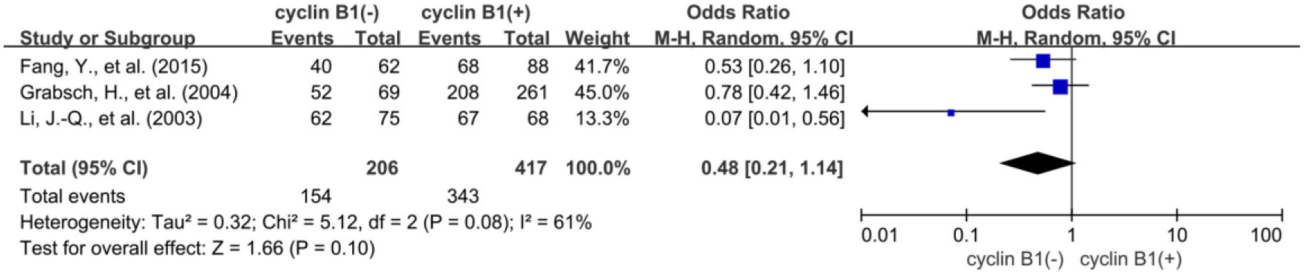

Supplementary Figure S1: Subgroup analysis of 3-year OS by cyclin B1 expression in colorectal cancer.

Study or Subgroup	cyclin B1(-) Events	cyclin B1(-) Total	cyclin B1(+) Events	cyclin B1(+) Total	Weight	Odds Ratio M-H, Random, 95% CI
Begnami, M. D., et al. (2010)	92	231	52	229	60.6%	2.25 [1.50, 3.38]
Kim, D.-H. (2007)	1	3	16	20	39.4%	0.13 [0.01, 1.75]
**Total (95% CI)**		**234**		**249**	**100.0%**	**0.72 [0.05, 11.51]**
Total events	93		68			

Heterogeneity: Tau² = 3.26; Chi² = 4.51, df = 1 (P = 0.03); I² = 78%
Test for overall effect: Z = 0.23 (P = 0.82)

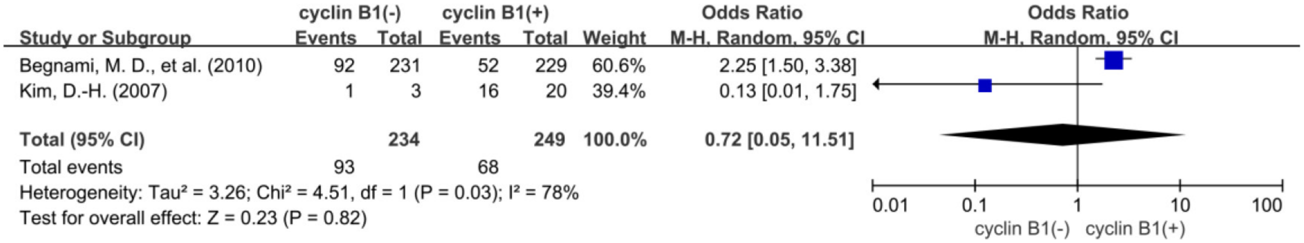

Supplementary Figure S2: Subgroup analysis of 5-year OS by cyclin B1 expression in gastric cancer.

Study or Subgroup	cyclin B1(-) Events	cyclin B1(-) Total	cyclin B1(+) Events	cyclin B1(+) Total	Weight	Odds Ratio M-H, Random, 95% CI
Arinaga, M., et al. (2003)	53	100	52	74	35.1%	0.48 [0.25, 0.90]
Kim, D.-H. (2007)	1	3	14	20	11.2%	0.21 [0.02, 2.84]
Soria, J. C., et al. (2000)	23	60	3	17	23.7%	2.90 [0.75, 11.20]
Suzuki, T., et al. (2007)	53	63	35	46	29.9%	1.67 [0.64, 4.34]
**Total (95% CI)**		**226**		**157**	**100.0%**	**0.97 [0.35, 2.69]**
Total events	130		104			

Heterogeneity: Tau² = 0.66; Chi² = 9.31, df = 3 (P = 0.03); I² = 68%
Test for overall effect: Z = 0.05 (P = 0.96)

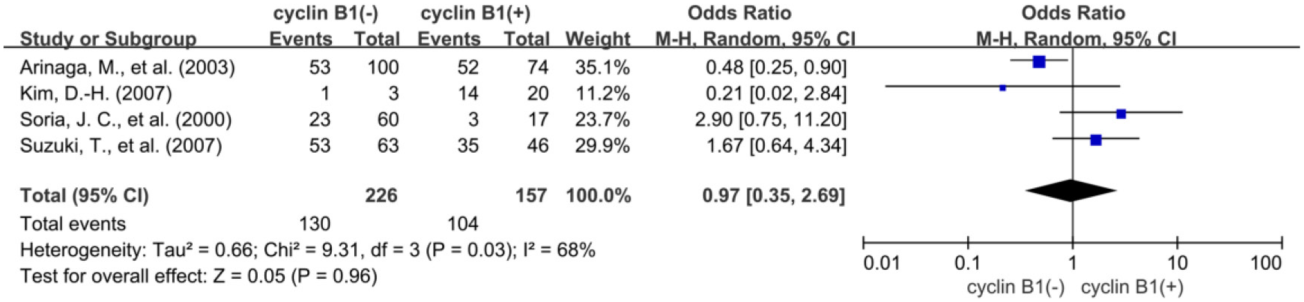

Supplementary Figure S3: 10-year OS by cyclin B1 expression.

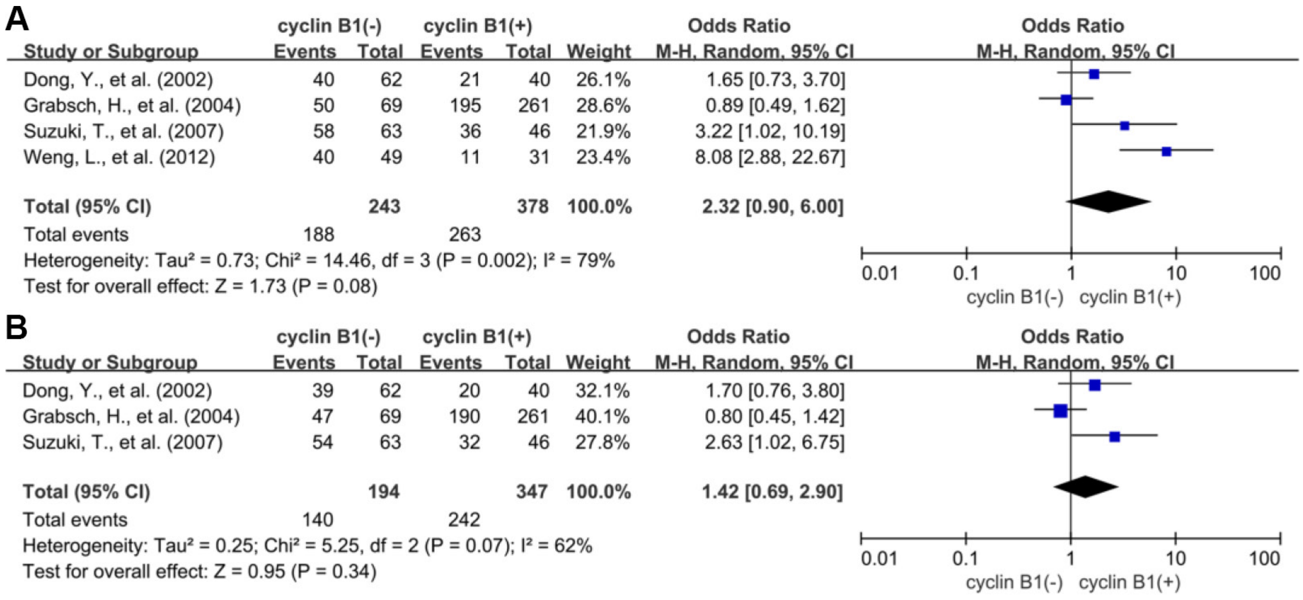

Supplementary Figure S4: 3- and 5-year DFS by cyclin B1 expression. (**A**) 3-year DFS; (**B**) 5-year DFS.

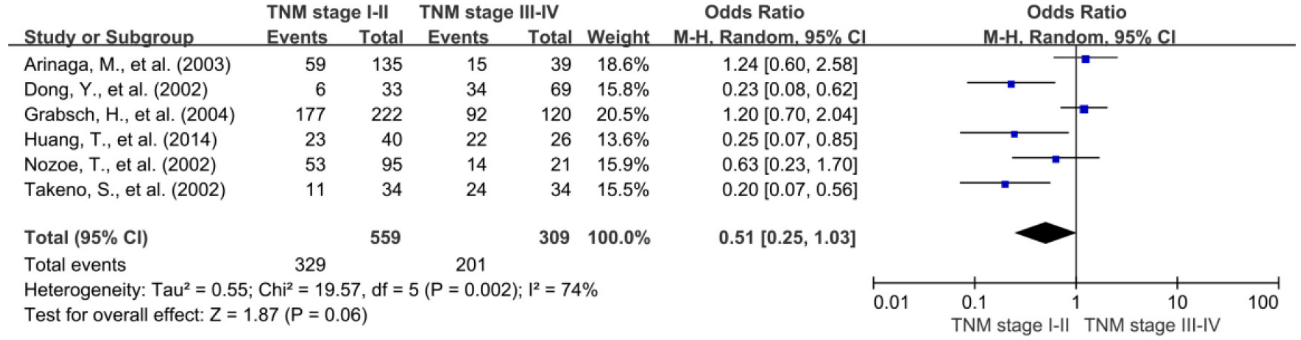

Supplementary Figure S5: Subgroup analysis the association of cyclin B1 expression and TNM stage.
